# Vertical Transmission, Risk Factors, and Antimicrobial Resistance Patterns of Group B *Streptococcus* among Mothers and Their Neonates in Southern Ethiopia

**DOI:** 10.1155/2022/8163396

**Published:** 2022-07-11

**Authors:** Belayneh Regasa Dadi, Mulatu Sime, Mohamed Seid, Dagimawie Tadesse, Munira Siraj, Dagninet Alelign, Zerihun Solomon

**Affiliations:** ^1^Department of Medical Microbiology, Arba Minch University, Arba Minch, Ethiopia; ^2^Madda Walabu University, Robe, Ethiopia; ^3^Dilla University, Dilla, Ethiopia

## Abstract

**Background:**

Group B *Streptococcus* (GBS) contributes to maternal and neonatal morbidity and mortality by increasing intrauterine infection or vertical transmission at the time of birth. Despite many efforts to reduce the potential risk of vertical transmission, GBS remains the main cause of serious disease (neonatal sepsis, meningitis, and/or pneumonia) in vulnerable newborns during the first week of life. This study aimed to assess vertical transmission, risk factors, and antimicrobial resistance patterns of GBS among pregnant women and their neonates.

**Methods:**

A facility-based cross-sectional study was conducted among mothers and their neonates from February to May 2021. A total of 201 pregnant women with their neonates participated in this study. A well-designed questionnaire was used to collect sociodemographic and clinical data. A vaginal swab from mother before delivery and neonatal nasal and ear canal swab samples were taken as soon as after delivery within 30 minutes. Vaginal swabs, neonatal ear canal, and nasal swabs were placed into Todd–Hewitt broth and incubated at 37°C for 18–24 hours at 35–37°C in 5% CO_2_ conditions and then subcultured on 5% sheep blood agar for 18–48 hours. Presumptive identification of GBS was made by morphology, Gram stain, catalase, and hemolytic activity on sheep blood agar plates. CAMP and bacitracin susceptibility tests were used as confirmatory tests for GBS. Data were analyzed using SPSS version 21. *P* value ≤0.05 was considered statistically significant.

**Results:**

Vertical transmission rates of GBS (mother to neonates) were 11.9%. The prevalence of GBS among pregnant women and newborns was 24/201 (11.9%) (95% CI = 7.5–16.9) and 11/201 (5.5%) (95% CI = 2.5–9.0), respectively. The history of prolonged rupture of membranes (AOR = 3.5, CI = 2.2–18.8) and urinary tract infection (AOR = 2.9, CI = 1.7–16.3) were associated factors for maternal GBS colonization. Gestational age of <37 weeks (*p*=0.008), low birth weight of <2.5 kg (*p*=0.001), and maternal history of vaginal discharge (*p*=0.048) were associated factors for neonatal GBS colonization. Low antibiotic resistance was observed for erythromycin 8.6%, clindamycin 5.7%, and chloramphenicol 2.9%.

**Conclusion:**

In this study, high vertical transmission (mother to neonates) rate was observed. The prevalence of vaginal GBS colonization of women at delivery was 11.9% and significantly associated with the history of prolonged rupture of membranes and urinary tract infections. Gestational age of <37 weeks, low birth weight of <2.5 kg, and maternal history of vaginal discharge were associated with neonatal GBS colonization. Hence, there is a need for antenatal culture-based GBS screening, risk factor-based interventions, and regular follow-up of drug resistance patterns for proper treatment and management of GBS.

## 1. Introduction

Globally, newborn mortality is a critical problem resulting in over 3 million deaths per year with 2/3rd occurring in the first week of life. Neonatal sepsis and pneumonia are responsible for 20% of those deaths [1]. Group B *Streptococcus* (GBS) is among the leading causes of early onset sepsis (EOS) worldwide with a 12% global case-fatality rate, which can be 3 times higher in low-income countries [2]. The burden of GBS early onset disease extends beyond neonatal illness and death, including such long-term impairments as vision or hearing loss and mental retardation. Over half (50%) of the survivors of bacterial meningitis diagnosed with GBS develop lifelong neurological sequelae such as increased intracranial pressure, stroke, development of cerebral palsy, blindness, deafness, epilepsy, and educational deficits in later life [3].

Group B *Streptococcus* colonization of the genitourinary or gastrointestinal tract of pregnant women and its transmission to the infant during the labor and delivery process is the principal risk factor for early onset invasive GBS disease [4]. During pregnancy, approximately 10–30% of women are colonized with GBS in the vagina and rectum asymptomatically as normal flora and 60% of their infants acquire this organism through the birth canal. About 30–70% of colonized mothers deliver GBS-colonized newborns and 1-2% of these develop early onset infections where heavily colonized mothers are more likely to transmit GBS to their offspring [5, 6]. Infection of the fetus can occur either by an invasion of amniotic fluid following prolonged rupture of membranes (PROM) or through direct acquisition during birth [6, 7]. The listed worldwide “classical” risk factors for vertical transmissions are as follows: GBS bacteriuria at any time during pregnancy, chorioamnionitis, PROM (>18 hours), prematurity (<37 weeks), prolonged labor (>12 hours), and intrapartum fever (≥38°C). Preterm infants are at 3-fold to 30-fold greater risk of developing early onset disease (EOD) compared with full-term infants, with the highest risk at lower gestational ages [3, 8, 9].

Universal screening of mothers for vaginal or rectal GBS colonization at 35–37 weeks of gestation and a risk-based strategy for selective intrapartum antibiotic prophylaxis (IAP) for all screen-positive women are the strategies currently recommended by CDC [1]. *Streptococcus agalactiae* remain fully susceptible to penicillin as well as to most *β*-lactams, and penicillin remains the first-choice antibiotic to prevent GBS-EOD and to treat GBS disease. However, over the last two decades, resistance to macrolides and clindamycin among invasive isolates of GBS has increased from <5% to common resistance of 20–30% [5,10]. GBS is associated with maternal and neonatal morbidity and mortality which is a major health concern; understanding the vertical transmission, risk factors and antimicrobial resistance patterns among mothers and neonates are very important for proper treatment and management of GBS since these data are lacking in the study area.

## 2. Methods

### 2.1. Study Design, Setting, and Population

A facility-based cross-sectional study was conducted from February to July 2021 at Arba Minch General Hospital, Southern Ethiopia. Study populations in this study were all pregnant women at gestation and attending Arba Minch General Hospital for delivery and their neonates. An inclusion criterion was women who are with live born. Exclusion criteria include women who used antibiotics within 2 weeks of labor, physically or mentally incapable of responding, assigned for caesarian section, and presented with obstructed labor, hemorrhage, severe preeclampsia, or fetal distress. Vertical transmission indicates mother-neonate GBS colonization during labor (delivery) or neonatal GBS colonization from GBS colonized mother. Vertical transmission at birth was confirmed by culture of swabs taken from the ear canal and nose of a neonate as soon as possible after delivery within 30 minutes.

### 2.2. Data Collection

Sociodemographic and clinical data were collected with a pretested well-designed questionnaire. Clinical/obstetrical characteristics such as gravidity, prenatal care, parity, prolonged labor, premature rupture of membrane, gestational age, prematurity, maternal fever, low birth weight, and some underlying medical conditions such as gestational diabetes mellitus and HIV were collected from pregnant mothers. Vaginal swabs were collected within 30 minutes of delivery from the mother and neonate.

### 2.3. Laboratory Investigations

Vaginal swabs were collected from mothers by brushing the lower one-third of the vagina with sterile cotton-tipped swabs and swabs from neonates' nasal area and ear canal were collected within 30 minutes after birth following standard CDC guidelines for sample collection [11]. Sample swabs were placed in GBS broth (enrichment broth and transport medium) and transported to the Medical Microbiology and Parasitology Laboratory of Arba Minch University College of Medicine and Health Science for microbiological investigations.

Vaginal swabs, neonatal ear canal, and nasal swabs were placed into Todd–Hewitt broth (THB) (bioMerieux SA, France) supplemented with gentamicin (8 *μ*g/ml) and nalidixic acid (15 *μ*g/ml) (BioMerieux, France) and incubated at 37°C for 18–24 hours. If there was growth, it was subcultured on 5% sheep blood agar at 37°C for 18–48 hours. Presumptive identification of GBS was made by morphology, Gram's stain, catalase, and hemolytic activity on sheep blood agar plates. All Gram-positive and catalase-negative cocci isolates were further tested for CAMP and bacitracin susceptibility to confirm the isolate was GBS [11].

Antimicrobial susceptibility testing was performed based on the Kirby–Bauer disc diffusion method using Clinical and Laboratory Standard Institute (CLSI) guidelines [12]. The inoculums were prepared by suspending 4-5 isolated colonies of the same morphology in 5 ml of sterile 0.85% NaCl solution (saline) equal to 0.5 McFarland standards used as a reference to adjust the turbidity of bacterial suspensions. Swabs were inoculated on Mueller–Hinton agar plates supplemented with 5% defibrinated sheep blood. Antibiotic disks were placed and incubated at 35–37°C under 5% CO_2_ atmosphere for 20–24 hours. The following antimicrobials were used with their respective concentration: penicillin (10 IU), ampicillin (10 *μ*g), erythromycin (15 *μ*g), azithromycin (15 *μ*g), clarithromycin (15 *μ*g), vancomycin (3 *μ*g), clindamycin (2 *μ*g), tetracycline (30 *μ*g), ceftriaxone (30 *μ*g), and chloramphenicol (30 *μ*g). The zones of inhibition were measured in millimeters using a ruler or caliper. The sizes of inhibition zones were graded according to the CLSI as sensitive, intermediate, or resistant to each antibiotic tested [12].

### 2.4. Data Quality Assurance

A pretest was conducted on 5% of study participants. Standard operational procedures were prepared and followed strictly. Culture media was prepared as per the manufacturer's instruction, and sterility was checked by incubating representative of the batch at 35–37°C overnight and observing bacterial growth. Those batches of culture media that showed growth was discarded. Control strains of *S. aureus* (ATCC 25923), *E. coli* (ATCC 25922), and *S. agalactiae* (ATCC 12386) were used.

### 2.5. Statistical Analysis

Data were entered and analyzed using the Statistical Package for Social Science (SPSS) version 21 (IBM statistics, Armonk, NY). The degree of association between dependent and independent variables was assessed using an adjusted odds ratio with a 95% confidence interval. A *p* value of ≤0.05 was considered as statistically significant.

## 3. Results and Discussion

### 3.1. Baseline Sociodemographic Characteristics

The age of the study participants ranged from 17 to 40 years with a mean age of 24.96 SD ± 4.33. A majority (54.2%) of women participants' age was greater than 25 years (Table 1).

### 3.2. Prevalence of GBS among Pregnant Women and Their Newborns

The prevalence of GBS among pregnant women and neonates was 24/201 (11.9%) (95% CI = 7.5–16.9) and 11/201 (5.5%) (95% CI = 2.5–9.0), respectively. The overall GBS colonization rate in both pregnant women and neonates was 35/402 (8.7%). In our study, the prevalence of GBS among pregnant women was 11.9%. This finding is comparable with studies conducted in Ethiopia [13–17], Brazil [18], Japan [19], and Germany [20]; but it is lower than studies done in Tanzania [21] and Zimbabwe [22]. However, it is higher than studies done in Cameroon [23], Mozambique [24], and China [25]. The difference in prevalence may be due to geographical differences, sample size, and methodological variations [17, 26].

The overall vertical transmission rate of GBS from colonized pregnant women to their newborns was 11/24 (45.8%) (Table 2). The proportion of vertical transmission of GBS from pregnant mother to newborn in our study was 45.8%, which is within the range of global reports of 40–70% [27]. However, it is higher than studies conducted in Ethiopia [17, 28] and Bangladesh [29]. These variations might be due to variations in geographic location, methodological variation, and service availability.

### 3.3. Clinical Factors Associated with Maternal GBS Colonization

The history of prolonged rupture of membranes (AOR = 3.5, CI = 2.2–18.8) and urinary tract infection (AOR = 2.9, CI = 1.7–16.3) were associated factors for maternal GBS colonization (Table 3). These findings are similar to studies conducted in Sri Lanka [30], India [31], and Korea [32].

### 3.4. Clinical Factors Associated with Neonatal GBS Colonization (Vertical Transmission)

Gestational age of <37 weeks (*p*=0.008), low birth weight of <2.5 kg (*p*=0.001), and maternal history of vaginal discharge (*p*=0.048) were associated factors for neonatal GBS colonization (Table 4). These findings are in line with studies done in India [9], Indonesia [33], Korea [32], and Guatemala [2]. Thus, investigating the risk factors which might be associated with vertical transmission would be useful for preventive strategies [26].

### 3.5. Antimicrobial Susceptibility Patterns

In this study, low antibiotic resistance was observed for erythromycin 8.6%, clindamycin 5.7%, and chloramphenicol 2.9%. Most GBS isolates were susceptible to antibiotics (Table 5). The result of erythromycin resistance is comparable with reports from Ethiopia [34], Tanzania [21], and Brazil [35]. Clindamycin resistance is comparable with reports from Tanzania [21], Brazil [35], and Kuwait [36]. The widespread use of these antibiotics to prevent early onset GBS disease has raised concern about the development of antibiotic resistance among GBS isolates [17, 37]. The drug resistance level underlines the need of carrying out antimicrobial susceptibility testing before prescription of antibiotics.

## 4. Conclusion

In our study, a high vertical transmission (mother to neonates) rate was observed. The prevalence of GBS among pregnant women and their newborns was 11.9% and 5.5%, respectively. The history of prolonged rupture of membrane and history of maternal UTI were associated factors of maternal GBS colonization. Gestational age of <37 weeks, low birth weight of <2.5 kg, and maternal history of vaginal discharge were associated factors for neonatal GBS colonization. This study showed no drug resistance against penicillin as it is still the antibiotic of choice for treating GBS infections in Ethiopia and the first-line agent for intrapartum antibiotic prophylaxis. Low antibiotic resistance was found for erythromycin (8.6%), clindamycin (5.7%), and chloramphenicol (2.9%) which needs attention. Hence, there is a need for antenatal culture-based GBS screening, risk factor-based interventions, and regular follow-up of drug resistance patterns for proper treatment and management of GBS.

### 4.1. Limitation of the Study

Serotyping, minimal inhibitory concentration of antimicrobials, and molecular characterization of GBS isolates were not performed because of budget constraints.

## Figures and Tables

**Table 1:** Sociodemographic characteristics of pregnant women.

Variables	Variable's categories	Frequency	Percentage (%)
Age group	<25	92	45.8
	≥25	109	54.2

Residence	Urban	122	60.7
	Rural	79	39.3

Marital status	Married	200	99.5
	Divorce	1	0.5

Occupation	Student	32	15.9
	Civil servant	35	17.4
	Housewife	118	58.7
	Merchant	16	8.0

Educational status	Unable to read and write	62	30.8
	Primary school	47	23.4
	Secondary school	43	21.4
	Tertiary education	49	24.4

**Table 2:** Status of GBS in mothers and neonatal body site swab samples.

Category	Frequency	Percentage (%)
Status of GBS in vaginal swab samples of mothers (*n* = 201)		
Positive	24	11.9
Negative	177	88.1
Total	201	100

Status of GBS in the neonatal ear canal and nasal swab samples (*n* = 201)		
Positive	11	5.5
Negative	190	94.5
Total	201	100

Overall GBS colonization rates (*n* = 402)		
Overall colonization rate	35/402	8.7
Vertical transmission rate	11/24	45.8

**Table 3:** Multivariate analysis of clinical factors with GBS colonization among pregnant women.

Variables	Variable's categories	Frequency	GBS result		COR (95% CI)	*P* value	AOR (95% CI)	*P* value
			Positive	Negative				
Gravidity	Primigravida	80	9 (11.3)	71 (88.7)	0.9 (0.37–2.16)	0.806		
	Multigravida	121	15 (12.4)	106 (87.6)	1			

Parity	Nullipara	25	9 (36.0)	16 (64.0)	4.7 (1.7–13.2)	0.003	1.7 (0.42–6.5)	0.469
	Primipara	73	4 (5.5)	69 (94.5)	0.49 (0.2–1.6)	0.232		
	Multipara	103	11 (10.7)	92 (89.3)	1			

Gestational age	<37 weeks	10	5 (50)	5 (50)	9.1 (2.4–34.13)	0.001	4.4 (.45–19.1)	0.259
	≥37 weeks	191	19 (9.9)	172 (90.1)	1			

History of UTI	Yes	13	8 (53.3)	7 (46.7)	12.1 (4.0–37.8)	≤0.001	2.9 (1.7–16.3)	0.008
	No	188	16 (8.6)	170 (91.4)	1			

History of PROM	Yes	46	15 (32.6)	31 (67.4)	7.9 (3.2–19.6)	≤0.001	3.5 (2.2–18.8)	0.001
	No	155	9 (5.8)	146 (94.2)	1			

Contraceptives used	Injectable	48	7 (14.6)	41 (85.4)	1.14 (0.39–3.3)	0.812		
	Injection and pills	41	2 (4.9)	39 (95.1)	0.34 (0.1–1.7)	0.184		
	Loop	37	6 (16.2)	31 (83.8)	1.3 (0.4–4.0)	0.656		
	None	69	9 (13.0)	60 (87.0)	1			

Number of ANC visited	0–3 times	63	8 (12.7)	55 (87.3)	1.1 (0.5–2.8)	0.823		
	4-5 times	138	16 (11.6)	122 (88.4)	1			

Previous still birth	Yes	8	2 (25)	6 (75)	2.59 (0.5–13.6)	0.261		
	No	193	22 (11.4)	171 (88.6)	1			

Previous abortion	Yes	27	5 (18.5)	22 (81.5)	1.85 (0.63–5.47)	0.263		
	No	174	19 (10.9)	155 (89.1)	1			

Vaginal discharge	Yes	13	6 (42.9)	8 (57.1)	7.0 (2.2.-22.6)	0.001	3.0 (0.62–14.6)	0.173
	No	188	18 (9.6)	169 (90.4)	1			

AOR, adjusted odds ratio; CI, confidence interval; COR, crude odds ratio; PROM, prolonged rupture of membranes; UTI, urinary tract infection; *n*, sample size.

**Table 4:** Multivariate analysis of clinical factors with GBS colonization (vertical transmission) among neonates.

Variables	Variable's categories	Frequency	GBS result		COR (95% CI)	*P* value	AOR (95% CI)	*P* value
			Positive	Negative				
Gravidity	Primigravida	80	6 (7.5)	74 (92.5)	1.9 (0.55–6.39)	0.311		
	Multigravida	121	5 (4.1)	116 (95.9)	1			

Parity	Nullipara	25	5 (20.0)	20 (80.0)	6.2 (1.53–25.09)	0.011	0.9 (0.10–8.42)	0.938
	Primipara	73	2 (2.7)	71 (97.3)	0.7 (0.12–3.91)	0.682		
	Multipara	103	4 (3.9)	99 (96.1)	1			

Gestational age	<37 weeks	10	5 (50)	5 (50)	30.8 (7.0–135.8)	≤0.001	2.2 (1.9–5.4)	0.008
	≥37 weeks	191	6 (3.1)	185 (96.9)	1			

Maternal UTI	Yes	13	6 (40.0)	9 (60.0)	24.1 (6.2–94.3)	≤0.001	3.6 (1.7–14.3)	0.145
	No	188	5 (2.7)	181 (97.3)	1			

History of PROM	Yes	46	8 (17.4)	38 (82.6)	6.8 (1.89–24.32)	0.003	5.07 (0.95–22.7)	0.056
	No	155	3 (1.9)	152 (98.1)	1			

Duration of PROM	<18 hours	181	5 (2.8)	176 (97.2)	15.1 (4.1–55.7)	≤0.001	3.28 (0.51–20.95)	0.210
	>18 hours	20	6 (30.0)	14 (70.0)	1			

Birth weight	<2.5 kg	11	5 (45.5)	6 (54.5)	25.6 (6.1–107.7)	≤0.001	2.5 (1.3–12.8)	0.001
	≥2.5 kg	190	6 (3.2)	184 (96.8)	1			

Maternal fever	Yes	18	2 (11.1)	16 (88.9)	2.42 (0.48–12.16)	0.284		
	No	183	9 (4.9)	174 (95.1)	1			

Types of contraceptives used	Injectable	48	3 (6.3)	45 (93.7)	0.85 (0.19–3.75)	0.834		
	Injection and pills	41	1 (2.4)	40 (97.6)	0.32 (0.04–2.80)	0.306		
	Loop	37	2 (5.4)	35 (94.6)	0.73 (0.14–3.97)	0.717		
	None	69	5 (7.2)	64 (92.8)	1			

Number of ANC visit	0–3 times	63	2 (3.2)	61 (96.8)	0.47 (0.10–2.24)	0.343		
	4-5 times	138	9 (6.5)	129 (93.5)	1			

History of abortion	Yes	27	2 (7.4)	25 (92.6)	1.47 (0.30–7.18)	0.637		
	No	174	9 (5.2)	165 (94.8)	1			

Vaginal discharge	Yes	13	6 (42.9)	8 (57.1)	27.3 (6.9–48.7)	≤0.001	1.8 (2.6–6.6)	0. 048
	No	188	5 (2.7)	182 (97.3)	1			

AOR, adjusted odds ratio; CI, confidence interval; COR, crude odds ratio; PROM, prolonged rupture of membranes; UTI, urinary tract infection; *n*, sample size.

**Table 5:** Antimicrobial susceptibility patterns of GBS isolates from pregnant mothers and neonates.

Antimicrobial	Disc potency (*μ*g)	Susceptible, *N* (%)	Intermediate, *N* (%)	Resistant, *N* (%)
Ampicillin	10	35 (100)	0	0
Penicillin	10	35 (100)	0	0
Ceftriaxone	30	35 (100)	0	0
Vancomycin	30	35 (100)	0	0
Erythromycin	15	24 (68.6)	8 (23.0)	3 (8.6)
Azithromycin	15	35 (100)	(0)	0
Clarithromycin	15	34 (97.1)	1 (2.9)	0
Tetracycline	30	29 (82.9)	6 (17.1)	0
Chloramphenicol	30	31 (88.6)	3 (8.6)	1 (2.9)
Clindamycin	2	26 (74.3)	7 (20.0)	2 (5.7)
Ciprofloxacin	5	35 (100)	0	0

## Data Availability

The datasets generated and/or analyzed during the current study are not publicly available due to ethical and confidentiality reasons but are available from the corresponding author upon request.

## Abbreviations

AOR: Adjusted odds ratio
CAMP: Christie, Atkins, and Munch–Peterson
CDC: Center for Disease Control and Prevention
CI: Confidence interval
CLSI: Clinical and Laboratory Standards Institute
COR: Crude odds ratio
EOD: Early onset diseases
EOS: Early onset sepsis
IAP: Intrapartum antibiotic prophylaxis
GBS: Group B *Streptococcus*
PROM: Prolonged rupture of membranes
TBH: Todd–Hewitt broth.
